# Efficacy of a Diode Vaginal Laser in the Treatment of the Genitourinary Syndrome of Menopause

**DOI:** 10.3390/bioengineering10101158

**Published:** 2023-10-02

**Authors:** Marta Barba, Alice Cola, Desirèe De Vicari, Clarissa Costa, Arianna Petra Castelli, Silvia Volontè, Robert Fruscio, Matteo Frigerio

**Affiliations:** 1 Department of Gynecology, IRCC San Gerardo dei Tintori, University of Milano-Bicocca, 20900 Monza, Italy; m.barba8792@gmail.com (M.B.); alice.cola1@gmail.com (A.C.); d.devicari@campus.unimib.it (D.D.V.); c.costa14@campus.unimib.it (C.C.); s.volonte6@campus.unimib.it (S.V.); robert.fruscio@unimib.it (R.F.); 2 Department of Gynecology, ASST Lecco, Manzoni Hospital, 23900 Lecco, Italy; a.castelli35@campus.unimib.it

**Keywords:** genitourinary syndrome of menopause, quality of life, sexual dysfunction, pelvic floor disorders, diode laser

## Abstract

Introduction: Genitourinary syndrome of menopause (GSM) and vulvovaginal atrophy (VVA) are the most frequent menopause-related clinical entities and are consistently included in the definition of pelvic floor disorders (PFDs). Nonhormonal therapies, such as lubricants and moisturizers, are indicated as first-line treatments, while the “gold standard’’ is represented by topical estrogen products; however, in cancer survivors hormonal treatment is not indicated. For this reason, energy-based therapeutic approaches—for instance, through laser technologies—may be employed as alternative options in this kind of patient; however, there are no studies evaluating the efficacy of a pure diode vaginal laser in the treatment of GSM. As a consequence, with our study, we aimed to evaluate outpatient nonablative diode laser treatment in sexually active women, with contraindications, no response, or refusal of local estrogenic therapy. Methods: This prospective study included patients with GSM, aged ≥ 18 years old, with contraindications, or refusal of local estrogen therapies. Women were evaluated via the use of their Vaginal Health Index (VHI) scores, which consists of five measures: elasticity, fluid volume, pH, epithelial integrity, and moisture. Moreover, the intensity of VVA symptoms (vaginal burning, vaginal itching, vaginal dryness, dyspareunia, and dysuria) was measured using a 10 cm visual analog scale (VAS), where the left extreme of the scale (score = 0) indicated “absence of symptom” and the right indicated “symptom as bad as it could be” (score = 100). Sexual function was evaluated with the Female Sexual Function Index (FSFI-19) questionnaire. The treatment was performed using a Leonardo Dual diode laser (Biolitec Italia Srl, Milano, Italy). The laser treatment consisted of three sessions, one per month. One month after the third session, the VHI, symptom VAS, and FSFI-19 were re-evaluated. In addition, the Patient Global Impression of Improvement (PGI-I) questionnaire was collected. Results: Our study enrolled a total of 26 consecutive patients. All patients were either in menopause or under treatment with gonadotropin-releasing hormone (GnRH). None of the patients reported adverse effects after laser treatment. In total, 19 (73.1%) patients referred improvements of their symptoms according to PGI-I scores. All domains of the FSFI-19 questionnaire, significantly improved after the diode laser treatment. The mean VHI score increased by 3.2 points, from 12.2 to 15.4 (*p* < 0.001). Additionally, we documented a significant improvement in symptoms affecting the VAS score, from 69.2 to 43.5 points (*p* < 0.001). Conclusion: A diode vaginal laser is an effective and easily tolerated ambulatory procedure for vaginal functional restoration in the treatment of GSM and VVA.

## 1. Introduction

Pelvic floor disorders (PFDs) constitute a comprehensive expression encompassing various conditions that jeopardize the female continence mechanisms, including urinary and fecal functions, as well as pelvic organ support. Pelvic floor disorders (PFDs) comprise various conditions, including pelvic organ prolapse, bowel dysfunction, and bladder dysfunction. These conditions typically arise due to the weakening of or damage to the pelvic floor, often associated with obstetric trauma [1,2]. PFDs share common risk factors and often co-occur or recur [3,4], such as age, menopausal status, and obesity [1,5]. Moreover, alterations in the composition of connective tissue and the presence of metalloproteinases have been documented in individuals with PFDs [6]. Additionally, PFDs can develop or persist as a result of prior pelvic floor surgeries [7,8].

Notably, they are a prevalent issue in women, particularly as they age [9]. Traditionally, the definition of PFDs in women has remained confined to urinary incontinence (UI) and pelvic organ prolapse (POP); however, this limited definition does not underline the significant role played by the pelvic floor in female sexual function and other bladder-related disorders that may not necessarily result in incontinence. Furthermore, it does not account for the distinct influence of hormones on the pelvic floor. In a more extensive scope, especially concerning menopausal women, an exhaustive definition of PFDs should encompass overactive bladder (OAB) syndrome and coital dysfunction. Menopausal coital dysfunction primarily arises due to atrophic changes, which manifest in symptoms such as vaginal dryness and dyspareunia. Simultaneously, atrophic changes within the lower urinary tract contribute to OAB symptoms, including dysuria and recurrent urinary tract infections (UTIs). Collectively, these atrophic symptoms are recognized as genitourinary syndrome of menopause (GSM), a term introduced by the North American Menopause Society in 2014, highlighting the profound hormonal influence on genitourinary and pelvic floor function. In its most comprehensive definition, menopausal PFDs encompass UI, POP, and conditions associated with GSM [10]. GSM and vulvovaginal atrophy (VVA) are the most frequent menopause-related clinical entities. During fertile years, the female genital apparatus maintains its trophism due to the hormonal stimulus of both estrogens and progesterone; after menopause the entire genital tract becomes atrophic. As estrogen levels decrease, whether naturally or due to hormone-altering treatments for malignancies, various alterations occur in the vaginal wall. These include the thinning of the vaginal lining, reduced vascularity and elasticity, shifts in vaginal secretions’ quality and quantity, and a loss of collagen fibers [6]. These changes, often associated with menopause, result in heightened tissue fragility and an elevated risk of vaginal and urinary infections, irritation, dryness, urogenital discomfort, urinary leakage, vaginal tissue damage, and a general deterioration in the quality of life; typically, these conditions tend to inevitably worsen if untreated [10,11,12,13,14,15,16]. Additionally, the female genitalia may experience vaginal relaxation syndrome (VRS), with the mucosa becoming weak and lax over the years; this circumstance can potentially result in stress urinary incontinence and diminished coital sensation. GSM embraces a spectrum of conditions, including VVA, urinary symptoms indicative of OAB, such as urgency, frequency, and nocturia, sterile dysuria, and recurrent UTIs. VVA is characterized by classical symptoms like vulvar pruritus, vulvar burning or pain, vaginal dryness, dyspareunia, and, in some instances, postcoital bleeding. Additionally, a subset of women may report vaginal discharge or malodorous sensations [17]. Recurrent UTIs are precisely defined as three uncomplicated, culture-confirmed infections occurring within the preceding 12-month period [18].

The treatment depends on symptoms, age, general health, and individual health risks [19]. First-line treatment consists of nonhormonal therapies, such as lubricants and moisturizers, while local estrogen products are considered the “gold standard’’. Newer hormonal therapeutic strategies may involve selective estrogen receptor modulators (SERMs); however, prior to prescribing a hormonal therapy regimen for survivors of breast or gynecological cancer, a comprehensive risk assessment must be conducted. This assessment should encompass variables such as patient age, treatment duration, dosage, therapy modality, administration route, malignancy histological subtype, and previous exposure history [20]. Moreover, in this peculiar population—due to pelvic irradiation, chemotherapy, or long-lasting hormonal therapy—symptoms may be more severe, persistent, and causing many consequences for sexual health and potentially even for patients’ adherence to therapy, which could significantly influence overall survival outcomes [20,21,22,23,24,25,26]. In these women, GSM and VVA are commonly managed using nonhormonal local interventions like hyaluronic acid, but their efficacy is moderately limited [27]; since safety data about estrogenic therapy are still limited it is not recommended in certain histologic types [20,28,29].

However, in cancer survivors energy-based therapeutic approaches—for instance, through laser technologies—may be employed as alternative options. An advantage of the more recent minimally invasive laser-based vaginal rejuvenation techniques, whether ablative or nonablative, is their potential to offer an alternative approach to addressing GSM/VVA. These methods have the capacity to enhance vascularization and all layers of connective tissue within the vaginal canal in a positive manner [30]. Multiple studies are emerging in the literature about the role of laser (light amplification by the stimulated emission of radiation) therapy, which may be routinely offered to these patients. Ablative laser devices, such as CO_2_ lasers, have been demonstrated to have great efficacy in mucosal rejuvenation; however, due to their mode of action, they may cause adverse effects like scars, infections, pigmentary alterations, and inflammation [31]. These complications may be reduced by improving physicians’ expertise and laser design or by opting for nonablative laser devices [32].

Specifically, nonablative devices might avoid the majority of these adverse effects since beams generate thermal damage directed only at the lower connective tissue, stimulating collagen production, while sparing the epithelium [33,34]. This type of thermal effect can be generated by diode lasers. A diode laser is an electronic device composed of two minute semiconductor materials, each measuring 50 μm. It produces a laser beam through the controlled passage of electrical current across a diode, regulated by a microprocessor, and subsequently transmitted to an optical fiber via an optical system, facilitating the delivery of light to the intended target location [35].

Preliminary data showed encouraging outcomes with hybrid lasers that partially involve the use of diode technology in patients with GSM [36]; however, to the best of our knowledge there are no studies evaluating the efficacy of a pure diode vaginal laser in the treatment of GSM. As a consequence, with our study we aimed to evaluate outpatient nonablative diode laser treatment in sexually active women, with contraindications, no response, or refusal of local estrogenic therapy. Specifically, we aimed to evaluate the efficacy and safety of laser therapy in patients reporting moderate to severe GSM symptoms after three sessions of a diode laser.

## 2. Materials and Methods

This study was conducted prospectively, with prior approval granted by the local ethics committee (protocol code: GSM-LASER). Recruitment took place between September 2022 and March 2023 from the gynecologic outpatients at Fondazione IRCCS San Gerardo dei Tintori in Monza, Italy.

The procedure to consider a patient eligible for the study included a clinical interview aimed at assessing the presence of sexual symptoms, contraindications, or no response or refusal of local estrogenic therapy. Patients were considered eligible if they have had at least two of the previously mentioned symptoms of GSM for at least one year, were aged ≥ 18 years old, had contraindications, or refused local estrogen therapies. None of the study’s tools were used as cutoffs as recruitment criteria.

Sociodemographic characteristics were collected at the baseline and inclusion/exclusion criteria were verified before starting the first laser application.

At the baseline, women were evaluated by using their Vaginal Health Index (VHI) scores, which consists of 5 measures: elasticity, fluid volume, pH, epithelial integrity, and moisture. The score ranges from 5 to 25, with a cutoff point at 15, where scores below this threshold indicate the presence of atrophic vaginitis [37]. This represents a semiobjective measure, considering that four out of the five variables—with the exclusion of vaginal pH measurement, calculated through a pH indicator strip—are influenced by practitioners’ judgments [38]. Moreover, the intensity of VVA symptoms (vaginal burning, vaginal itching, vaginal dryness, dyspareunia, and dysuria) was measured using a 10 cm visual analog scale (VAS), where the left extreme of the scale (score = 0) indicated “absence of symptom” and the right indicated “symptom as bad as it could be” (score = 100). Sexual function was evaluated with the Female Sexual Function Index (FSFI-19) questionnaire [39]. The FSFI-19 is a self-reported questionnaire employing a 5-point Likert scale, comprising 19 items distributed across six domains of sexual function: sexual desire, lubrication, arousal, orgasm, pain, and satisfaction. It stands out as a widely recognized and robust diagnostic instrument for assessing female sexual function and gauging treatment efficacy [40]. This scale has undergone validation in multiple languages and consistently exhibits strong psychometric attributes [39]. A proposed FSFI total score of 26.5 serves as the optimal threshold for distinguishing between women with and without sexual dysfunction [41].

**Figure 1:**
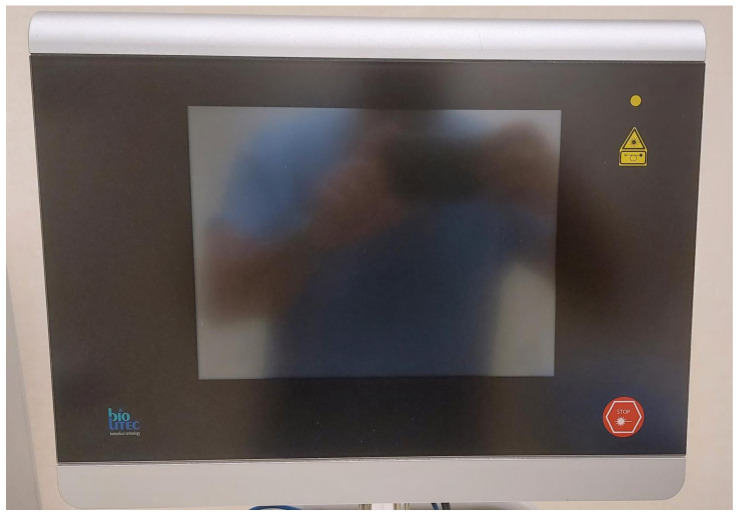
Leonardo Dual diode laser (Biolitec Italia Srl, Milano, Italy).

The treatment was performed using a Leonardo Dual diode laser (Biolitec Italia Srl, Milano, Italy) (Figure 1). This is a versatile diode laser that features the combination of two wavelengths, 980 nm and 1470 nm, offering a variety of tissue interactions. Laser treatment consisted of three sessions, one per month, following the manufacturer’s protocol. At each session the power was set to 7 watts in a pulsed mode (2 pulses of 0.5 s each, with 0.5 s of pause). After the administration of local lidocaine gel, the vaginal glass handpiece was inserted into the vagina, and the dedicated fiber was inserted into the handpiece. The procedure was performed in accordance with the producer’s recommendations. In total, 8 pulses were circumferentially delivered for every cm of the vagina, from the fornix to the introitus.

One month after the third session, the vaginal health index (VHI) was reassessed. Moreover, the intensity of VVA symptoms (measured by the VAS) and sexual function, measured with the FSFI-19, were re-evaluated. In addition, responses to the Patient Global Impression of Improvement (PGI-I) questionnaire were collected. The PGI-I questionnaire is a 7-point Likert scale that enables clinicians to evaluate the extent of improvement or deterioration in a patient’s condition relative to a baseline state established at the onset of treatment, using the following rates: 1, very much improved; 2, much improved; 3, minimally improved; 4, no change; 5, minimally worse; 6, much worse; or 7, very much worse [40].

Anonymized data were entered into the database by the authors. Statistical analysis was performed using JMP software version 9 (SAS Institute, Cary, NC, USA). Outcomes were reported as the mean ± standard deviation for continuous variables and as a number (percentage) for noncontinuous variables. Pre- and post-treatment comparisons were performed for objective as well as subjective outcomes and tested for statistical significance. Differences were tested using a paired T-test for continuous data and Fisher’s test for noncontinuous data. A *p*-value < 0.05 was considered statistically significant.

## 3. Results

In total, 26 patients completed the full treatment of three sessions of a diode vaginal laser in the period of interest. All patients were either in menopause or under treatment with gonadotropin-releasing hormone (GnRH). Five patients (19%) experienced pharmacological menopause as they were receiving GnRH, and five patients (19%) had a previous history of rectal or cervical cancer, so they underwent treatment with brachytherapy. We did not include these subgroups in our analysis as they constituted a small sample of our population.

**Figure 2:**
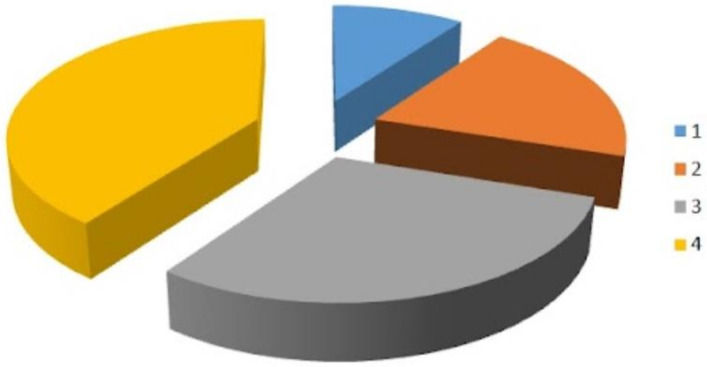
Patients’ improvements according to PGI-I scores. PGI-I score absolute values (1–4) are demonstrated.

The patients’ mean age was 57.6 ± 9.7 years, and the parity was 1.3 ± 1.1 births. The baseline findings of the FSFI-19, VAS, and VHI are shown in Table 1. None of the patients reported adverse effects after laser treatment. In total, 19 (73.1%) patients saw improvements in their symptoms according to the PGI-I scores (Figure 2). The FSFI-19, VAS, and VHI results after the treatment are reported in Table 2. All domains of the FSFI-19 questionnaire (desire *p* = 0.009, arousal *p* = 0.007, lubrication *p* = 0.010, orgasm *p* = 0.012, satisfaction *p* = 0.034, and pain *p* = 0.017), as well as the total score (*p* = 0.002), significantly improved after the diode laser treatment. The mean VHI score increased by 3.2 points, from 12.2 to 15.4 (*p* < 0.001). Moreover, we observed a significant improvement in symptoms affecting the VAS score, from 69.2 to 43.5 points (*p* < 0.001).

## 4. Discussion

VVA and GSM represent major health issues, taking into account the upward trend in the average age of women [38]. Moreover, physicians often show poor awareness regarding these genital pathologies, tending to consider the related symptoms as part of the common aging transition or side effects of cancer treatments [42]. This fact results in a relevant underestimation of the syndrome‘s prevalence. VVA must be considered as a progressive and chronic condition that often worsens without appropriate treatment or if therapy is suspended, due to its pathogenesis and connection to aging as well as menopause [38]. In cancer survivors, consequences for their quality of life may be worse, since menopausal symptoms appear at a younger age and are caused by a more rapid hormonal decline [43]. Considering the current limitations of the hormonal treatments for GSM in specific populations, other treatment options should be considered. Nonhormonal first-line therapies for GSM and VVA usually involve over-the-counter nonestrogenic vaginal emollients and lubricants, which may be able to alleviate genital symptoms in a usually only transitory way [44,45], since they do not reverse urogenital aging but only help to compensate for its anatomic and functional condition by improving vaginal secretions and sexual comfort [46]. Nonhormonal therapies also include energy-based devices, such as vaginal laser therapy, which represents a nonpharmacological second-line strategy. The use of laser technologies for the treatment of VVA has been increasing over the last few years [47]. This was due to the necessity to provide a therapeutic alternative for all those women who have no benefits, contraindications, or are not compliant with the other available first-line or hormonal therapies. A diode laser device, as well as other nonablative technologies, is based on the concept of photothermal effect. It induces the controlled heating of the collagen-rich layers of the vaginal wall, which stimulates fibroblast activity for collagen production. Consequently, collagen synthesis is enhanced, leading to an increase in collagen content within the vaginal tissues. The increased collagen content helps in restoring the structural integrity and elasticity of the vaginal mucosa. This tissue remodeling process reverses some of the atrophic changes associated with VVA, such as thinning and the loss of elasticity as well as vascularization [48].

Our study demonstrated that a diode vaginal laser is a safe and effective option in GSM patients, reducing the burden of symptoms, ameliorating quality of life, and improving sexual function. Specifically, 73.1% of patients in our study reported a subjective improvement in their symptoms according to PGI-I scores and the VAS. Similarly, a significant improvement in all domains of sexual life was demonstrated by the increase in FSFI-19 scores. From an objective point of view, a mean increase in the VHI score by 3.2 points was observed after three vaginal laser sessions.

Our findings are consistent with previous studies on the efficacy of a vaginal laser on GSM symptoms. The prevailing laser modalities presently employed in the therapeutic management of VVA encompass a fractional microablative CO_2_ laser and a nonablative photothermal Erbium: YAG laser [38]. Numerous clinical studies in the existing literature examined the effectiveness and safety of both of these devices implemented for the treatment of VVA. A meta-analysis examined the efficacy of CO_2_ laser treatment in postmenopausal women with VVA, in which 12 articles and 459 participants were included. In comparison to the initial baseline measurements, the VHIs exhibited statistically significant increases at the 1-month, 3-month, 6-month, and 12-month follow-up assessments (*p* < 0.001). Concerning the severity of VVA symptoms, specifically the VAS scores for vaginal dryness at the 1-month, 3-month, 6-month, and 12-month follow-up evaluations (*p* < 0.05), as well as for vaginal itching, burning, and dysuria at the 1-month follow-up (*p* < 0.001), and for dyspareunia at the 1-month, 3-month, 6-month, and 12-month follow-up assessments (*p* < 0.001), all demonstrated statistically significant reductions. For FSFI, the total scores at the 1-month, 3-month, 6-month, and 12-month follow ups (*p* < 0.001), and the scores in all of the domains at the 1-month follow up (*p* < 0.05), were all significantly higher [48]. Similarly, various studies have been conducted to evaluate the role of an Er: YAG Smooth laser (VEL) in the treatment of GSM and vaginal relaxation syndrome. Barber et al. identified the impact on and level of satisfaction of 40 women who were treated with VEL: 78% of the patients showed clinical improvement, while the degree of satisfaction was greater than 90% [49]. A prospective study by Bojanini et al. compared the effects of the VEL treatment on 40 women who attained natural or iatrogenic menopause. Before treatment, symptoms of VVA were similar in both groups: all participants presented with severe vaginal dryness and dyspareunia; however, after three months of VEL treatment, a substantial 70% of patients reported the absence of vaginal dryness, while 90% of patients reported no more symptoms of dyspareunia [50].

A diode laser is a recent technological advancement in the scenario of vaginal laser devices for the treatment of GSM symptoms. This represents a technological advancement in the nanoscience field for an emerging generation of medical technologies that are small-sized and more affordable than older laser devices [51]. Progressively, the use of laser devices has also concerned other gynecological procedures; in particular, a diode laser has been introduced in both hysteroscopic and laparoscopic surgery, demonstrating feasibility and safety in hysteroscopic metroplasty as well as polypectomy and in the laparoscopic dissection of deep endometriosis. In all of these procedures high-precision cutting, the accurate and consistent regulation of tissue vaporization, mastered power of penetration and deepening, a lack of electrical disturbances, low thermal dispersion, high safety, and good toleration were the most remarked advantages of this technology [35,52,53]; however, to the best of our knowledge this is the first study evaluating the efficacy of a diode vaginal laser in the treatment of GSM. The diode laser treatment was effective in improving sexual function and reducing VVA-related symptoms in women with GSM. Consequently, this may represent a valid and promising new alternative to other devices, such as CO_2_ and Er: YAG pieces of equipment. Other strengths of our study involve the prospective design and assessment of the advantages, including multiple validated questionnaires. Limitations, on the other hand, are represented by the small sample size, the lack of a control group, and the limited follow-up. Moreover, since there were no previous studies on diode lasers for GSM, data on expected outcomes were not available, and thus a power calculation was not performed.

## 5. Conclusions

A diode vaginal laser is an effective and easily tolerated ambulatory procedure for vaginal functional restoration in the treatment of GSM and VVA. This technology has been demonstrated to be minimally invasive, safe, and effective in reducing clinical symptoms and improving patients’ quality of life. As a consequence, it represents a valid option in reducing GSM and VVA symptoms in those patients with contraindications, no response, or refusal of local estrogenic therapy.

## Figures and Tables

**Table 1:** Baseline Female Sexual Function Index (FSFI-19), Vaginal Health Index (VHI), and symptom VAS.

Total FSFI-19 Score	11.0 ± 8.5
- Desire domain	3.8 ± 1.6
- Arousal domain	6.0 ± 5.7
- Lubrication domain	5.8 ± 5.8
- Orgasm domain	4.2 ± 4.7
- Satisfaction domain	6.0 ± 4.4
- Pain domain	3.5 ± 4.2
VHI score	12.2 ± 2.8
Symptom VAS	69.2 ± 25.9

**Table 2:** Comparison before (T0) and after (T1) the vaginal laser. FSFI-19: Female Sexual Function Index; VHI: Vaginal Health Index; PGI-I: Patients Global Impression of Improvement; and n/A: not applicable.

	T0	T1	*p*-Value
Total FSFI-19 Score	11.0 ± 8.5	15.7 ± 9.5	0.002
- Desire domain	3.8 ± 1.6	4.7 ± 2.0	0.009
- Arousal domain	6.0 ± 5.7	8.9 ± 5.7	0.007
- Lubrication domain	5.8 ± 5.8	8.4 ± 6.5	0.01
- Orgasm domain	4.2 ± 4.7	6.2 ± 5.3	0.012
- Satisfaction domain	6.0 ± 4.4	7.4 ± 4.5	0.034
- Pain domain	3.5 ± 4.2	5.6 ± 5.5	0.017
VHI score	12.2 ± 2.8	15.4 ± 3.6	<0.001
Symptom VAS	69.2 ± 25.9	43.5 ± 27.6	<0.001
PGI-I	n/A	2.8 ± 0.9	n/A

## Data Availability

The data presented in this study are available on request from the corresponding author.
